# Cartridge syringe vs computer controlled local anesthetic delivery system: Pain related behaviour over two sequential visits – a randomized controlled trial

**DOI:** 10.4317/jced.52542

**Published:** 2015-10-01

**Authors:** Yogesh-Kumar Thoppe-Dhamodhara, Sharath Asokan, Baby-John John, GeethaPriya Pollachi-Ramakrishnan, Punithavathy Ramachandran, Praburajan Vilvanathan

**Affiliations:** 1 Senior Lecturer, Department of Pedodontics and Preventive Dentistry, KSR Institute of Dental Science and Research, Tiruchengode, TamilNadu, India; 2 Professor, Department of Pedodontics and Preventive Dentistry, KSR Institute of Dental Science and Research, Tiruchengode, TamilNadu, India; 3 Professor and Head of Department, Department of Pedodontics and Preventive Dentistry, KSR Institute of Dental Science and Research, Tiruchengode, TamilNadu, India; 4 Reader, Department of Pedodontics and Preventive Dentistry, KSR Institute of Dental Science and Research, Tiruchengode, TamilNadu, India

## Abstract

**Background:**

Local anesthetic injection is one of the most anxiety provoking procedure in dentistry. Knowledge about change in pain related behaviour during consecutive visits helps in and scheduling of treatment procedures and management of children in dental clinic.

**Aim:**

To compare the pain perception, behavioural response and the associated change in physiological parameters while receiving local anesthesia injection with cartridge syringe and computer controlled local anesthetic delivery system (CCLAD) over two consecutive visits.

**Material and Methods:**

In this randomized controlled cross over trial, 120 children aged 7 – 11 years were randomly divided into group A: receiving injections with CCLAD during first visit; group B: receiving injections with cartridge syringe during first visit. The physiological parameters (heart rate and blood pressure) were recorded before and during injection procedure. Objective evaluation of disruptive behaviour and subjective evaluation of pain perceived were done using Face Legs Activity Cry Consolability (FLACC) scale and modified facial image scale (FIS) respectively.

**Results:**

No statistical difference in pain response (*p*= 0.164) and disruptive behaviour (*p* = 0.120) between cartridge syringe and CCLAD injections were seen during the first visit although the latter showed lesser scores. However, during the second visit there were significant increase in pain response (*p* = 0.004) and disruptive behaviour (*p* = 0.006) in cartridge syringe group with an associated increase in heart rate.

**Conclusions:**

Injections with CCLAD produced lesser pain ratings and disruptive behaviour than cartridge syringe in children irrespective of order of visit.

** Key words:**Behaviour, cartridge syringe, CCLAD, local anesthesia.

## Introduction

Pain is a complex multidimensional phenomenon affected by mostly psychological and physiological factors (1). Dental visits can become more difficult due to the anticipated pain especially if an injection is expected. This can lead to uncooperative behaviour, especially in children and delay the treatment (2). When painful stimuli like injections are repeated over time, different reaction trends are possible (3). Pain related behaviour may either increase in the successive appointments or a habituation to the painful stimulus may occur.

Local anesthetic injection is one of the most commonly used agent for pain reduction. Unfortunately, the pain reducing anesthetic agent cannot be administered in a 100% pain-free method. Topical gel/spray application, use of thinner needles, cartridge syringe injections, jet injections and computer controlled local anesthetic delivery (CCLAD) systems have been used to minimise this discomfort. Asarch T *et al.*, Gibson *et al.*, Allen KD *et al.*, Ram D *et al.*, Lopez *et al.*, Tahmassebi *et al.*, Langthasa M *et al.* have compared the pain response during local anesthetic delivery with cartridge and CCLAD system (4-10). Very few studies have compared the influence of visits on pain related behaviour while receiving injections with cartridge syringe and CCLADs. Versloot *et al.* found no significant differences in pain and distress response between CCLAD and traditional syringe over two consecutive visits (11). Hembrecht *et al.* showed that the children displayed more disruptive behaviour during second treatment visit while receiving injections with two types of computerized devices (1). The results obtained were contradictory and not conclusive. Hence this randomised controlled trial was planned to compare pain perception, behavioural response and the associated change in physiological parameters while receiving local anesthesia injection with cartridge syringe and computer controlled local anesthetic delivery system (CCLAD) over two consecutive visits.

## Material and Methods

This randomized controlled trial with cross over design was carried out in the Department of Pedodontics and Preventive Dentistry. The study protocol was approved by the institutional review board and ethical committee consent (ref 011/KSRIDSR/EC/2011) was obtained. Written consent was obtained from parents of participating children.

-Inclusion and exclusion criteria

One hundred and twenty children were included in the study based on the following inclusion criteria: a) Age 7 to 11 years; b) Children with ASA (American Society of Anesthesiologists) I status; c) No previous history of dental treatment who needed at least 2 clinical sessions of operative procedures preceded by local anaesthetic injection, one on either side of the maxilla or mandible, neither of which was due to emergency. Exclusion criteria were: a) children allergic to local anesthetics (lidocaine); b) children under medications that could alter the pain perception; c) medically compromised and special children; d) uncooperative patients (Frankl behaviour rating 1 – definitely negative).

-Randomization

The children were randomly divided into 2 groups: Group A – receiving injections with CCLAD during first visit and then cartridge injections; Group B – receiving injections with cartridge (conventional) during first visit followed by CCLAD injections. Randomization pattern was generated using computer software.

-Measurement of baseline data

Before commencement of the treatment procedure, pulse oximeter probe (FTP -101, SCure Pvt Ltd, Gujarat, India) and blood pressure cuff of digital blood pressure monitor (Omron Healthcare Pvt Ltd, Singapore) were fixed on the right hand index finger and on the left arm respectively. The baseline data of heart rate and blood pressure were obtained in the counselling room 10 minutes before procedure with the patient seated on a chair in an erect position. Three readings were taken and the mean score was calculated.

-Equipment used

**Figure 1:**
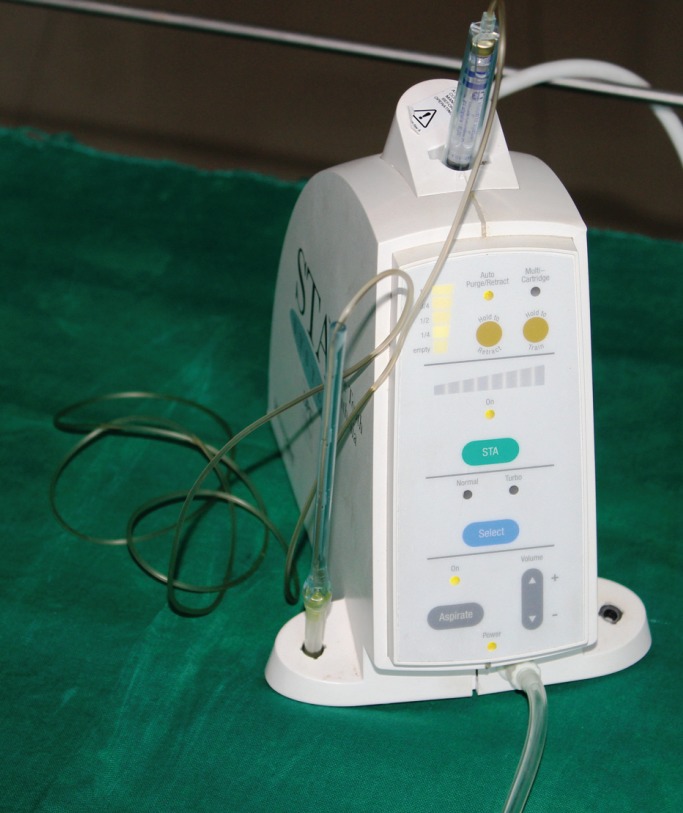
CCLAD with handpiece.

The CCLAD (STA Wand, Milestone Scientific Pvt Ltd, Livingston, USA) delivers the anesthetic as at uniform pressure irrespective of the tissue resistance due to its Dynamic Pressure Sensing TechnologyTM (DPS). The anesthetic can be delivered in 3 modes: Single tooth Anesthesia (STA) (1cc per 207 seconds), normal (1cc per 35 seconds), and turbo (1cc per 17 seconds) which can be selected from the display on the front panel of device. The rate of local anesthetic delivered is also controlled by pressure activated foot control pedal attached to the CCLAD. Various gauges (27, 28, and 30) and length (0.5 inch, 0.75 inch, 1 inch, 1.25 inch) of needles are available for different injection techniques. It is equipped with indicator lights to display and audible signals that monitor the cartridge volume, pressure at tip of needle, aspiration mode (Fig. 1).

-Injection procedure and interpretation

**Figure 2:**
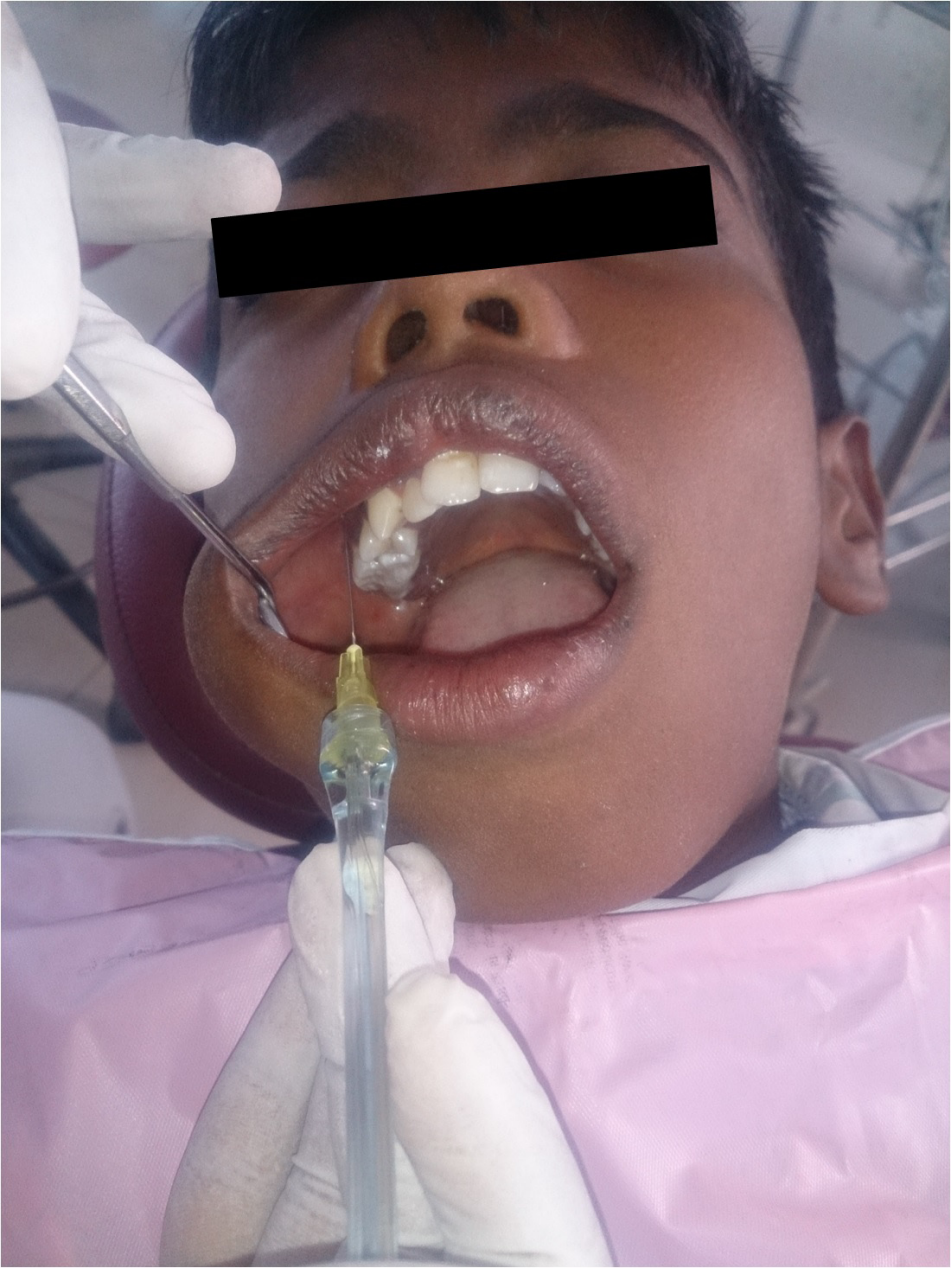
Injection with CCLAD.

Children were familiarized with the interpretation of modified Facial Image Scale (FIS) after being seated on the dental chair. The injection procedure was explained to the children using standard and similar euphemisms. The injection site was dried with cotton and topical anesthetic gel was applied and allowed to remain for 30 seconds. Two percent lidocaine with 1:1,00,000 epinephrine was then administered with a 1 inch 30 gauge needle using birotational technique to minimize needle deflection (12). Injections with CCLAD were given in Single Tooth Anesthesia (STA) mode initially till 1/4th of cartridge was administered followed by the normal mode. Injections with cartridge syringe were given slowly at approximately 1ml/min with an aspirating cartridge syringe (Septodont, France). All the injections were given by the same operator /primary investigator, to ensure that the results were not influenced by inter-operator variability. Objective evaluation of disruptive behaviour was done using FLACC scale by a calibrated dental assistant. The physiological parameters (heart rate, blood pressure) were recorded during the injection procedure. Subjective evaluation of pain was rated using a modified Facial Image Scale after the injection procedure. The washout period between the visits was 1 week (13). During the next appointment the child was administered local anesthetic injection using the alternative technique on other side of the jaw (Fig. 2).

-Statistical analysis

The data obtained were statistically analysed using SPSS software (15.0, SPSS Inc., Chicago Ill, USA). t test, Mann Whitney test were for comparing mean scores of FIS, FLACC of both modes of local anesthetic administration. Paired t test and Wilcoxon signed rank test were used to compare the quantitative data of a single group over two time periods *p* ≤ 0.05 was considered as statistically significant.

## Results

One hundred and twenty children, 71 boys and 49 girls (mean age = 9.23 ± 1.52 years) were included in the study. The attrition rate was 4.5% (n = 10) as they did not report for the second appointment. One hundred and ten children were subjected to both computerized and conventional (cartridge syringe) injection technique.

There were no significant difference on comparing FIS (*p* = 0.164) and FLACC (*p* = 0.120) scores of children receiving cartridge syringe and CCLAD injections during first visit ( Table 1). There were no significant differences in physiological parameters ( Table 2). During second visit there was a significant increase in FIS (*p* = 0.004) and FLACC (*p* = 0.006) scores in children receiving cartridge syringe injections ( Table 1). Heart rate showed a significant increase (*p* = 0.007) while receiving injections with cartridge syringe ( Table 3). On comparing the FIS and FLACC scores of children receiving injections with cartridge syringe on first and second visit there were no significant differences (*p* > 0.05) among them. The physiological parameters too showed no significant differences. Similar results were obtained on comparing the first and second visit scores while receiving CCLAD injections ( Table 4).

**Table 1:**
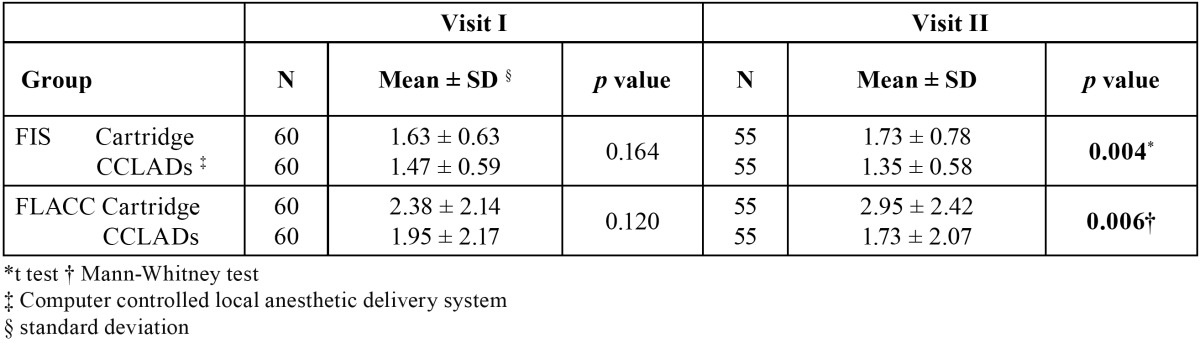
Comparison of mean FIS and FLACC scores in I and II visit of cartridge and CCLADs injections.

**Table 2:**
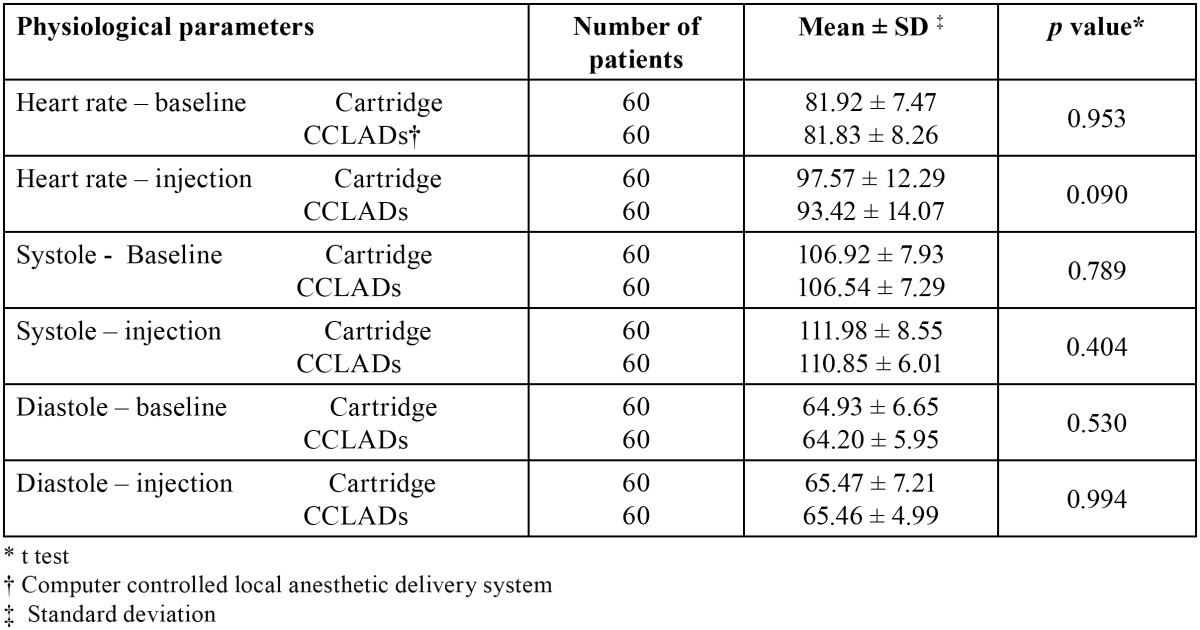
Comparison of physiological parameters in I visit cartridge and CCLADs injections.

**Table 3:**
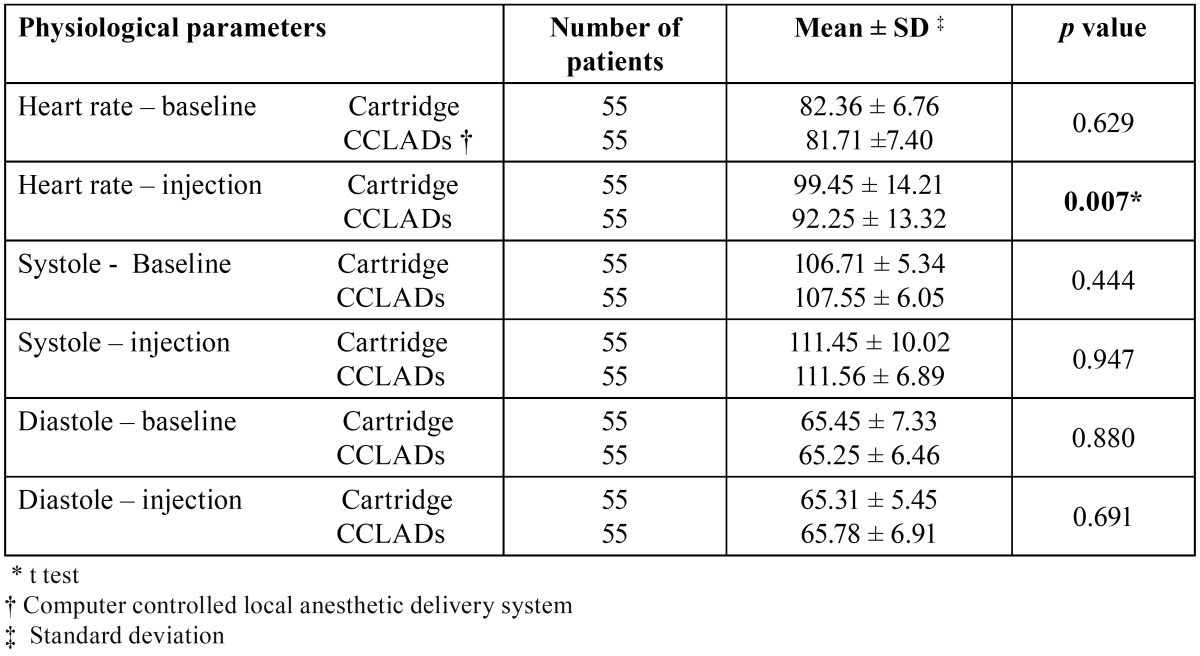
Comparison of physiological parameters in II visit of cartridge and CCLADs injections.

**Table 4:**
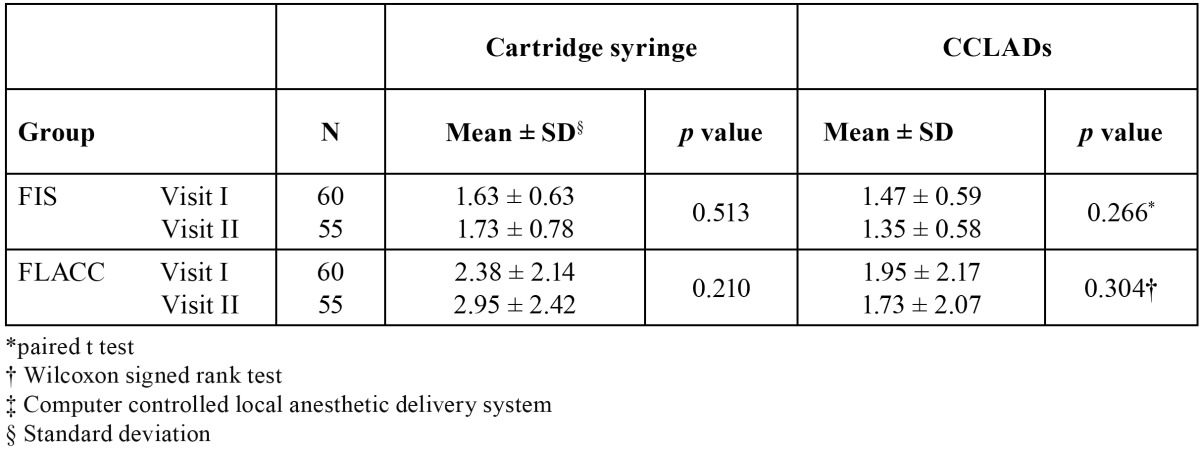
Mean FIS and FLACC scores in I and II visit of cartridge and CCLAD injections.

## Discussion

Literature search showed discrete data regarding the influence of visits on the behavioural response to different injection techniques. Hence this randomised controlled cross-over trial was planned to assess various factors like pain perception, behavioural response and physiological parameters in children during local anesthetic administration with cartridge syringe and CCLAD in consecutive visits.

The children included belong to concrete operational period (7-11 years) of Jean Piaget’s cognitive theory. They are capable of logical reasoning when the problem is displayed before them and this helps them in making decisions. These children have a cognition which is adult-like and the subjective nature common in younger children reduces as the cognition continues to develop.

In the present study cross over design was followed where the children served as his/her control. This was in accordance with the studies done by Ram D *et al.*, Palm AM *et al.*, Langthasa M *et al.* (7,10,14) and in contrast to studies by Asarch T *et al.*, Gibson RS *et al.*, Tahmassebi JF *et al.* where a parallel design was followed (4,5,9). No attempt was made to sex match as there was no significant difference in pain reaction between girls and boys Ram D *et al.*, Tahmassebi JF *et al.* (7,9). The children were not blindfolded during injection procedure as followed by Asarch T *et al.*, Gibson RS *et al.*, Allen K *et al.* (4-6) as it can increase the anxiety of children during the dental procedures. Hence, in this study standard euphemisms and distraction techniques were used to reduce anxiety in both the groups.

Facial Image Scale is a valid and reliable measure of dental anxiety for employment with young children in clinical settings (15). Subjective evaluation of pain was done using a modified Facial Image Scale. Ideally a scale should be short in length to maximize response from children and minimize time for administration; easy to hold the attention of child and be simple to score and interpret (16). In this study the scale was modified to 3 faces signifying: a) no discomfort, b) mild discomfort, c) severe discomfort. This was done to reduce the confusion among children while assessing pain. The subjective evaluation may differ according to child’s pain threshold level. So FLACC scale was used for objective evaluation of the child’s behaviour during injection procedure. This evaluation provides more information about the discomfort the child is experiencing as it is measured during injection procedure rather than asking the child to rate his response after the injection. FLACC scale can be used for quantifying pain behaviours in children who cannot verbalize the presence or severity of pain. FLACC scale is a validated and a reliable scale used in assessing pain in acutely ill adults and children post general anesthesia (17). FLACC pain assessment tool incorporates five categories of pain behaviours: facial expression; leg movement; activity; cry; and consolability.

On comparing the pain and distress response of children receiving injections with cartridge syringe and CCLAD during sequential visits it was seen that there were no significant differences between them during the first visit although the latter showed lesser scores. The physiological parameters also followed the above trend. The insignificant results in the first visit could be due to anxiety, fear of unknown and bodily injury among the children. The mere anticipation of pain and intrusion into the oral cavity may result in lowering of pain threshold. According to Chapman HR, Kirby Turner NC, there are five factors which are important in the aetiology and perpetuation of dental fear: a) Fear of pain or its anticipation; b) A lack of trust or the fear of betrayal; c) Fear of loss of control; d) Fear of the unknown; e) Fear of intrusion (18).

In the second visit the children who received CCLAD injections showed significantly less disruptive behaviour than children receiving injection with cartridge syringe. This probably led to significantly increased heart rate as the injection pressure with cartridge syringe is difficult to control. Increased pain ratings were seen in cartridge group during the second visit inspite of the child being habituated to the injection procedure.

Versloot J *et al.* compared pain and distress response in children aged 4- 11 years who received injections with CCLAD and traditional syringe over two sequential dental visits. He showed that no significant differences could be found on injection with CCLADs and conventional syringe over first and second visits. However, during the first visit, highly anxious children showed more pain, distress and pain related behaviour than low anxious children. He concluded that level of anxiety was an important factor in the response of a children reaction to a local anesthetic injection (11).

There were no significant differences in facial image scores on comparing the usage of cartridge syringe in the first (1.64 ± 0.64) and second visit (1.73 ± 0.78). Disruptive behaviour during first (2.42 ± 2.21) and second (2.95 ± 2.42) visit also showed no significant differences. There were no significant differences in the physiological parameters too. Similar results were obtained for children receiving CCLAD injections. The present study results are in accordance to Ram D *et al.* who found no significant difference in the behaviour reaction of children when the CCLAD was delivered during first or second visit, within or between different age groups. She identified a trend that children who reacted negatively to one technique reacted the same way to another (7). Hembrecht *et al.* used two types of computerized device in preschool children and found increased pain related behaviour in the second treatment session particularly in high anxious children as they have less coping strategies compared to low anxious children. The high anxiety may be due to previous sensitisation to a dentist and exposure to actual treatment. The disruptive behaviour seen was not dependent on the type of type of computerized device used in first treatment session (1).

The results of this study cannot be generalised for the entire treatment procedure. The operator and subjects were not blinded to the mode of local anesthetic delivery. An attempt was made to minimize this bias by using an independent observer for coding the behaviours. Due to cross over nature of the study design, children were expected to show reduced anxiety during the second visit thus exhibiting lesser pain response and disruptive behaviour. However the results show that the FIS and FLACC scores of children receiving injections with cartridge were higher in the second visit. CCLAD injection scores were lesser during first and the second visit compared to the other mode.

The present study concluded that irrespective of the visit, injections with CCLAD produced lesser pain response and disruptive behaviour than cartridge syringe. Use of CCLAD can be considered as a possible step towards achieving a relatively pain-free pediatric dental practice and also in developing a positive attitude towards dental treatment.
